# Shear bond strength of ceramic bracket bonded to different surface-treated ceramic materials

**DOI:** 10.4317/jced.55330

**Published:** 2018-12-01

**Authors:** Niwut Juntavee, Apa Juntavee, Krittaphat Wongnara, Pimkhwan Klomklorm, Ronnaphum Khechonnan

**Affiliations:** 1 Department of Prosthodontics, Faculty of Dentistry, Khon Kaen University, Khon Kaen, Thailand; 2 Department of Pediatric Dentistry, Faculty of Dentistry, Khon Kaen University, Khon Kaen, Thailand; 3 Division of Biomaterials and Prosthodontics Research, Faculty of Dentistry, Khon Kaen University, Khon Kaen, Thailand

## Abstract

**Background:**

This study evaluated the effect of ceramic surface treatments on bond strength of ceramic brackets to machine-able ceramics and ceramic veneering metal.

**Material and Methods:**

Machined ceramic specimens (10x10x1.5 mm) were prepared from Empress® CAD (EP), and e.max® CAD (EM). Ceramic veneering metal specimens (PF) were fabricated from sintered d.Sign® porcelain (1.27 mm thickness) over d.Sign®10 metal (0.23 mm thickness). Each ceramic was divided into 3-groups and treated surface by Er-YAG laser (LE) or etching with 9.6% HF acid for 5 seconds (A5) or 15 seconds (A15). Resin adhesive (Transbond™-XT) was used for attaching ceramic brackets for each group (n=15) and cured with LED (Bluephase®) for 50 seconds. Specimens were immersed in distilled water for 24 hours before testing for shear bond at crosshead speed of 1.0 mm/min. The data were analyzed for the differences in bond strength with ANOVA and Tukey’s multiple comparisons (α = 0.05). De-bond surfaces were microscopically examined.

**Results:**

Bond strength (MPa) were 12.65±1.14 for EMA5, 14.50±2.21 for EMA15, 13.97±1.17 for EMLE, 12.40±1.95 for PFA5, 15.85±3.13 for PFA15, 14.06±2.17 for PFLE, 12.12±1.54 for EPA5, 15.65±1.57 for EPA15, 12.89±1.17 for EPLE. Significant differences in bond strength among groups were found related to surface treatment (*p*<0.05), but not significant difference upon type of ceramics (*p*>0.05). A15 provided higher bond strength than LE and A5 (*P*<0.05). No damage of ceramic surface upon de-bonding was indicated except for A15 tends to exhibit ditching. LE showed more uniform treated surface for bonding and no surface destruction upon de-bond compared to others.

**Conclusions:**

Bond strength was affected by surface treatment. Both LE and A15 treated surface provided higher bond strength than A5. Considering possibly inducing defect on ceramic surface, LE seems to provide better favorable surface preparation than others. Treated ceramic surface with Er-YAG prior to bracket bonding is recommended.

** Key words:**Ceramic, ceramic bracket, Er-YAG, laser, shear bond strength, surface treatment.

## Introduction

Ceramics have been widely utilized as restorative materials to repair damaged teeth in the form of veneers, crowns, and bridges due to their aesthetic property, high fracture resistance, and biocompatibility (1). Conventional silica-based ceramics consisting of feldspar and quartz are generally used in veneering metal for metal ceramic restoration. The increasing demand for aesthetic and precise restoration has led to the development of advanced ceramics materials for the fabrication of ceramic restorations based on digital technology. Ceramic restorations are nowadays more commonly found in adult orthodontic patients seeking treatment. There is an increasing likelihood that orthodontic brackets and attachments require to be placed on existing ceramic restorations. Ceramic material does not facilitate the bonding of bracket since the glazed surface of ceramic materials are inert for bonding with adhesive resin (2). Maintaining brackets on ceramic restorations can be problematic. Bonding failure rates have been reported to be 9.8% in 2 years (3). This is a considerably high failure rate compared to other adhesive procedures used in restorative dentistry. The retentive strength between resin adhesive and ceramic restoration was reported to be insufficient (4). Although conventional orthodontic banding can be employed instead of bonding, its results are unattractive, especially for anterior teeth, and it is not possible to place orthodontic bands on the bridge’s abutment. The bond strength of bracket to ceramic restoration must be adequate to withstand orthodontic force throughout the treatment period (5). It is crucial to apply an appropriate adhesive procedure that encourages sufficient bond strength during the course of orthodontic treatment and minimizes damage to ceramic restoration (6). It is also equally important that the restoration be free of damage and remain in the mouth after the de-bonding of the bracket at the end of the treatment (7). Thus, the adherence force should be sufficient to withstand bracket dislodgment throughout the treatment as well as offer feasibility in bracket removal without generating excessive force that can possibly create defect on the ceramic restorations (8).

Adherence force between orthodontic brackets and ceramics depends on many variables including the kind of bracket, ceramics, adhesive material, and method of ceramic surface treatment (9). Previous studies reported that the bond strength of either enamel or ceramic material to ceramic bracket is higher as compared to that of enamel or ceramic to metal bracket (10,11). Thus, it often exhibits ceramic surface damage upon ceramic bracket’s de-bonding. The different composition and crystalline structure of ceramic materials may require a different bonding technique to ensure sufficient adhesion of bracket to ceramic that is capable of withstanding orthodontic and masticatory forces during orthodontic treatment. This appears to be a problem as it is virtually impossible for clinicians to differentiate between various types of ceramic on existing restorations in clinical situations. Therefore, the procedure of bonding brackets to existing ceramic restorations requires consideration of an appropriate technique that ensures a durable bonding bracket and that the ceramic surface remains damage free after de-bonding (12). Since the inert property of ceramic surfaces does not facilitate adhesion through adhesive materials, several attempts were made to revolutionize the ceramic surface to promote adhesion through mechanical, chemical, or other combinations (13,14). The mechanical approach can involve roughening the ceramic surface by grinding it with diamond bur, sandpaper disc, or blasting with Al2O3 abrasives (15). However, these procedures produced a permanently destructive effect on the ceramic surface. The chemical approaches entail acid etching to provide bonding affinity to adhesive materials to adhere to ceramic restoration (16). Furthermore, the application of 9.5%–10% hydrofluoric acid (HF) was reported to be capable of creating irregularities on the ceramic surface, enabling micromechanical interlocking for resin adhesive (2,4,17,18). Extremely strong HF acid etching was required to produce a clinically acceptable bond strength; however, this method increased the risk of cohesive failure of ceramic during the de-bonding process and damaged the ceramic glazed surface.

Several lasers, such as neodymium-doped yttrium aluminum garnet (Nd-YAG), carbon dioxide (CO2), and erbium-doped yttrium aluminum garnet (Er-YAG), are being increasingly employed in dental practice for soft and hard tissue removal, cavity preparations, conditioning, and decontamination (19). Among them, Er-YAG is classified as a solid type laser that is appropriately utilized with hard dental tissue structure (20,21). The Er-YAG laser can produce an infrared range of 2,940 nm that can be absorbed by water and the OH-group of hydroxyapatite (22). Er-YAG has been used for the modification of enamel surface for bracket bonding (23). It appears to be suitable for ceramic surface modification for retaining adhesive resin because its energy emission is almost completely absorbed by the ceramic material (24,25). A few studies have investigated its capability in ceramic surface modification (26-28). However, no consensus has been reached in the literature with regard to Er-YAG treated ceramic’s ability to allow suitable bracket bonding (29).

Due to the advancement in the development of machinable ceramic material, the search for appropriate selection and manipulation for ceramic materials that can achieve sufficient bond strength between the ceramic bracket with different ceramic materials was conceptualized for this study. The aim was to compare the effects of different ceramic surface treatments on machinable ceramic restorative materials and conventional ceramic veneer metal on the shear bond strength of ceramic brackets. Two types of machinable ceramics, including IPS Empress® CAD (EP; Ivoclar Vivadent, Schaan, Liechtenstein) and IPS e.max® CAD (EM, Ivoclar Vivadent, Schaan, Liechtenstein) and one type of conventional metal ceramics (PF), using IPS d.SIGN® porcelain (Ivoclar Vivadent, Schaan, Liechtenstein) veneering to cast metal substructure (IPS d.sign® 10, Ivoclar Vivadent, Schaan, Liechtenstein) that were surface treated with three different methods, including Er-YAG lased (LE), HF etched for 5 seconds (A5), and HF etched for 15 seconds (A15), bonded to ceramic bracket (Inspire ICE™, Ormco, Orange, CA, USA) with adhesive resin (Transbond™ XT, 3M Unitek, St. Paul, MN, USA) were evaluated for shear bond strength ( Table 1). The null hypothesis was that Er-YAG lased surface of machinable ceramics and conventional ceramic veneer metal would result in comparable bond strength for ceramic bracket in relation to the HF etched surface for 5 and 15 seconds.

**Table 1:**
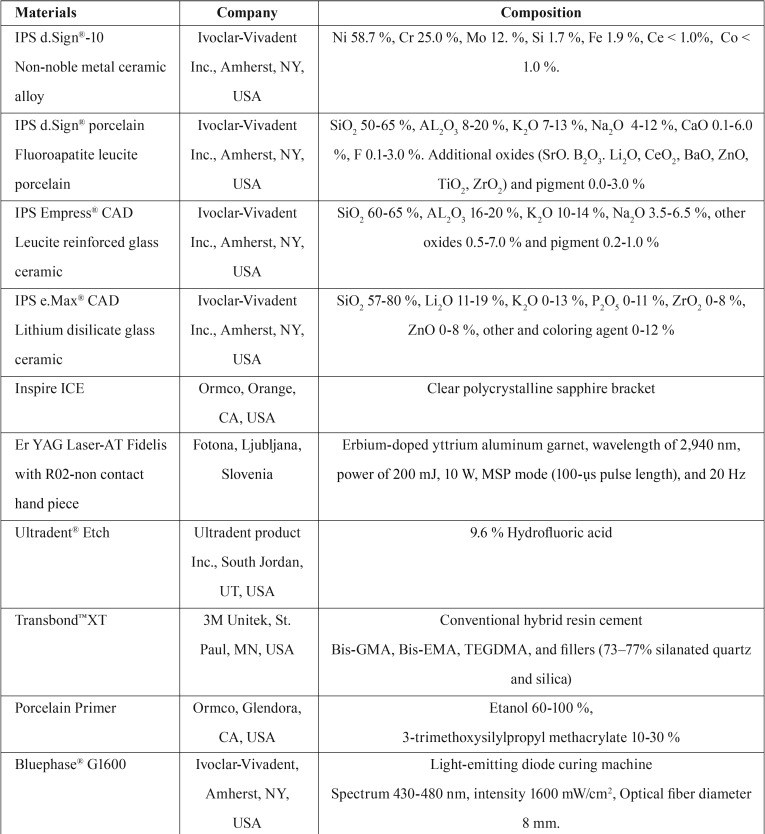
Materials, company and their compositions used in this study.

## Material and Methods

-Machinable ceramic specimen preparation

The IPS Empress® CAD (EP) and IPS e.max® CAD (EM) ceramic specimens (n = 50/each) were cut from the machinable ceramic blocks into square shape discs with the dimensions 10 × 10 × 1.7 mm (length x width x thickness) using a sectioning machine (Mecatome T180, Presi, Eybens, France). The ceramic specimens were polished to a series of 800, 1200, 2000, and 4000 abrasiveness of silicon carbide disc in the polishing machine (ECOMET® 3, Buhler, Lake Bluff, IL, USA). The diamond suspension (Metadi®, LakeBluff, IL, USA) was used to polish to obtain a smooth surface with the final dimensions of 10 × 10 × 1.5 mm (length x width x thickness). The EP specimens were then glazed, while the EM specimens were crystalized and glazed in the porcelain furnace (Programat® CS, Ivoclar-Vivadent, Schaan, Liechtenstein) following the manufacturer’s firing schedule at 850 °C for 1 minute.

-Metal ceramic specimen preparation

The conventional metal ceramic specimens (PF) (n = 50) were fabricated in a square-shaped disc. The metal discs of size 10 × 10 × 0.23 mm were casted, sandblasted with 50 microns aluminous oxide, and cleaned with distilled water in the ultrasonic machine. The opaque porcelain was applied to the metal surface, subsequently fired in a porcelain furnace according to the firing temperature recommended by the manufacturer. The opaque porcelain thickness of 0.27 mm needed to be achieved after firing for no more than two times. The dentine porcelain was condensed onto the fired opaque porcelain using a porcelain condenser (Shofu Co., Shiba, Japan) and fired in the porcelain furnace according to the manufacturer recommended firing schedule. The dentin porcelain thickness of 1.5 mm was produced upon firing for not more than twice. The body porcelain was polished and glazed according to the manufacturer’s recommendation at 850 °C for 1 minute to derive the final metal-ceramic disc dimension of 10 × 10 × 1.5 mm.

-Ceramic surface treatment 

The specimens in each group were cleaned in the ultrasonic cleaner (3M Unitek, St.Paul, USA) for 15 minutes to remove any surface residues and were then divided into three groups (15 samples each) for surface treatment with 3 different techniques, including HF etched for 5 seconds (A5), HF etched for 15 seconds (A15), and Er-YAG lased surface (L). The 9.5 % HF gel (Ultradent® Etch, Ultradent product Inc., South Jordan, UT, USA) was painted with a microbrush in the central area sized 4 x 4 mm of the ceramic for either 5 or 15 seconds, cleansed with spray water, and dried with an air-blower. The laser-treated groups (LE) were irradiated with Er-YAG laser (AT Fidelis, Fotona, Ljubljana, Slovenia) through a non-contact hand-piece (R02; 1.3 mm in diameter), at the power of 200 mJ, 10 W, 20 Hz, in MSP mode (100-ụs pulse length). A laser was lased perpendicular to the ceramic surface at the distance of 7 mm from the ceramic surface and in the central area of 4 x 4 mm with a water coolant for 20 seconds.

-Bonding bracket to ceramic treated surface

Each ceramic specimen was bonded with ceramic bracket (Inspire ICE) with adhesive resin (Transbond™ XT). The porcelain primer (Ormco, Glendora, CA, USA) was applied to the ceramic surface with a microbrush for 5 seconds and blown gently. The resin adhesive was introduced to the bracket’s base and firmly placed on the ceramic specimen with gentle force for approximately 5 N for 5 seconds to control the 25 micrometer of cement film thickness using a veneer caliper (Mitutoyo, Neuss, Germany). The excess cement was removed and then polymerized with a light-curing unit (Bluephase® G-1600, Ivoclar Vivadent, Schaan, Liechtenstein) for 50 seconds (10 seconds on each side and 10 seconds above the bracket). All samples were reserved in distilled water at 37 °C for 24 hours before testing.

-Evaluation of shear bond strength 

**Figure 1:**
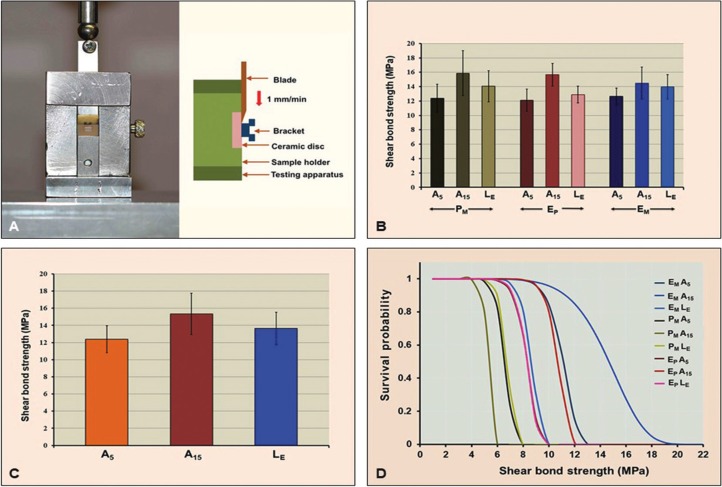
(A) Specimen was mounted in a testing jig and compressively load at the bracket-ceramic interface to determine for (B) shear bond strength of ceramic bracket to different ceramic materials upon different surface techniques, (C) significant differences in shear bond strength upon different surface treatment techniques were indicated, and (D) revealed Weibull survival probability of shear bond for each group.

The specimen was mounted in a custom made jig for testing in a universal testing machine (Lloyd Instruments Ltd., West Sussex, United Kingdom) as depicted in Figure 1(A). The load was vertically applied through the straight knife-edged chisel at the bracket-ceramic interface at a constant crosshead speed of 1.0 mm/min until bond failure happened. The loads at failure (P) were recorded and calculated for shear bond strength (σ) by dividing the failure load with the bracket base area (A), as illustrated in equation 1.

σ=P/A..........Equation 1

-Microscopic evaluation

The de-bonded bracket base and ceramic surface were examined visually under an optical stereomicroscope (Nikon, Melville, NY, USA) at 10 x magnification to ascertain the mode of failure (FM), ceramic surface damage index (CDI), and adhesive resin remnant index (ARI) (2,8,9). The FM was determined as follows.

Type I: Interfacial failure between bracket and adhesive resin (90% or more of bracket base was exposed and 10% or less of ceramic was free of adhesive).

Type II: Interfacial failure between adhesive resin and ceramic (10% or less of bracket base was exposed and 90% or more of ceramic was free of adhesive).

Type III: Failure of bracket (Fracture of bracket during removal, left part of bracket bonded on ceramic).

Type IV: Failure of ceramic (A portion of the ceramic was removed with the bracket base without loss of more than 10% of the adhesive from the bracket base).

Type V: Combination failure (Less than 90% but more than 10% of the bracket base was exposed or more than 10% but less than 90% of ceramic was free of adhesive).

The CDI was classified as follows:

0: No detectable ceramic surface damage. The surface remained intact, retaining the same condition as previous.

1: Localized detectable ceramic surface alteration limited to superficial surface observed under microscope.

2: Generalized detectable ceramic surface alteration limited to superficial surface observed under microscope;

3: Localized visually detectable ceramic surface damage, significantly repair required with composite resin.

4: Generalized visually detectable ceramic surface damage, significantly repair required with composite resin;

5: Localized ceramic surface damage or fracture.

6: Generalized ceramic surface damage of fracture.

The ARI were scored as follows.

0: no adhesive resin remained on ceramic;

1: ≤ 50% of adhesive resin remained on ceramic;

2: ≥ 50 % of adhesive resin remained on ceramic;

3: adhesive resin mostly remained on ceramic, showing an imprint of the bracket base.

The treated ceramic surface for each group was microscopically evaluated for different patterns of surface treatments. The samples were sputter coated with gold-palladium in a coating machine (K 500X, Emitech, Asford, UK) and examined with a scanning electron microscope (SEM, S-3000N, Hitachi, Tokyo, Japan) at x 5000 magnification.

-Statistical analysis

The data was statistically analyzed using SPSS/PC Version 20 software (IBM, Armonk, NY, USA). An analysis of variance (ANOVA) was employed to determine the significant effect of shear bond strength on different ceramics as well as ceramic surface treatment methods and their interactions. Post-hoc Tukey’s multiple comparison was determined for significant difference between each factor at 95% level of confidence. Weibull analysis was performed to evaluate the bond strength’s reliability using Weibull++®statistics (ReliaSoft, Tucson, AZ, USA), and estimated the Weibull modulus (m). A Chi-squared test was utilized to determine significant differences of the FM, CDI, and ARI in relation to each factor at 95% level of confidence.

## Results

The results of the shear bond strength tests have been described in  Table 2 and Figure 1(B). The highest bond strength was demonstrated in the group PFA15 (15.86 ± 3.13 MPa), followed by EPA15 (15.65 ±1.57 MPa), EMA15 (14.50 ± 2.21 MPa), PFLE (14.06 ± 2.17 MPa), EMLE (13.97 ± 1.17 MPa), EPLE (12.89 ± 1.17 MPa), EMA5 (12.65 ± 1.14 MPa), PFA5 (12.40 ± 1.95 MPa), and EPA5 (12.12 ± 1.54 MPa). The highest to lowest characteristic strength (σo, MPa) was indicated for the group PFA15, followed by EPA15, EMA15, PFLE, EMLE, EPLE, EMA5, PFA5, and EPA5 with the values 17.16, 16.35, 15.44, 15.01, 14.73, 13.61, 13.21, and 13.20 respectively, as presented in  Table 2. The ANOVA indicated significant difference in shear bond strength of ceramic bracket to ceramic materials as the effect of different surface treatment methods (*p* < 0.05) but revealed no significant different due to the type of ceramics and the interaction of two factors (*p* > 0.05), as indicated in  Table 3. The post hoc Tukey’s multiple comparisons indicated that the treatment surface of ceramic with A15 resulted in a significantly higher bond strength as compared to the treatment surface with LE, and A5 (*p* < 0.05). Both ceramic surface treatments with A15 and LE resulted in significantly higher bond strength in comparison to the surface reatment with A5 (*p* < 0.05), as shown in  Table 4 and Figure 1 (C). The Weibull modulus of shear bond strength, ranked from the highest to lowest, EMA5 (11.45), PFA15 (10.98), EMLE (8.89), PFA5 (8.58), PFLE (8.55), EMA15 (7.21), EPLE (6.94), EPA5 (6.80), and EPA15 (5.47), indicated a reliable survival probability of bond strength, as depicted in Figure 1 (D) and  Table 2.

**Table 2:**
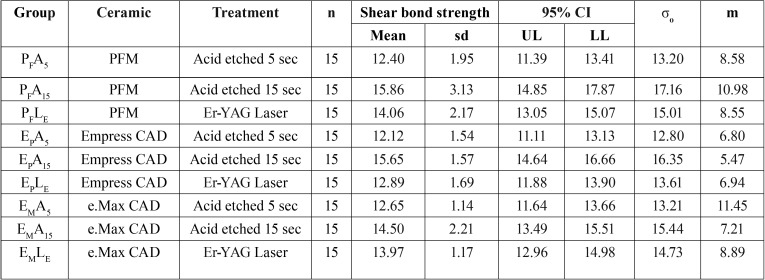
Mean, standard deviation (sd), 95% confidential interval (CI), characteristics strength (σo), and Weibull modulus (m) of shear bond strength of ceramic bracket bonded to porcelain fused to metal (PFM, PF), Empress CAD (EP), and e.Max CAD (EM) upon surface treated with either acid etched for 5 seconds (A5) or 15 seconds (A15), or Er-YAG laser (LE).

**Table 3:**
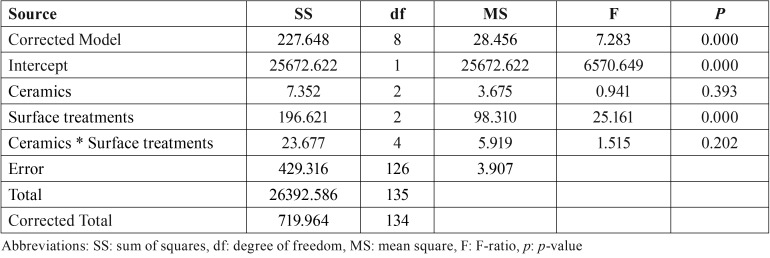
An analysis of variance (ANOVA) of shear bond strength of ceramic bracket bonded to porcelain fused to metal (PFM; PF), Empress CAD (EP), and e.Max CAD (EM) upon surface treated with either acid etched for 5 seconds (A5) or 15 seconds (A15), or Er-YAG laser (LE).

**Table 4:**
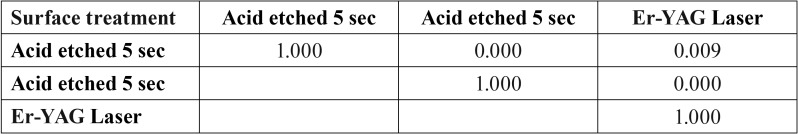
Post hoc Tukey’s multiple comparisons of shear bond strength of ceramic bracket bonded to porcelain fused to metal (PFM; PF), Empress CAD (EP), and e.Max CAD (EM) upon surface treated with either acid etched for 5 seconds (A5) or 15 seconds (A15), or Er-YAG laser (LE).

**Figure 2:**
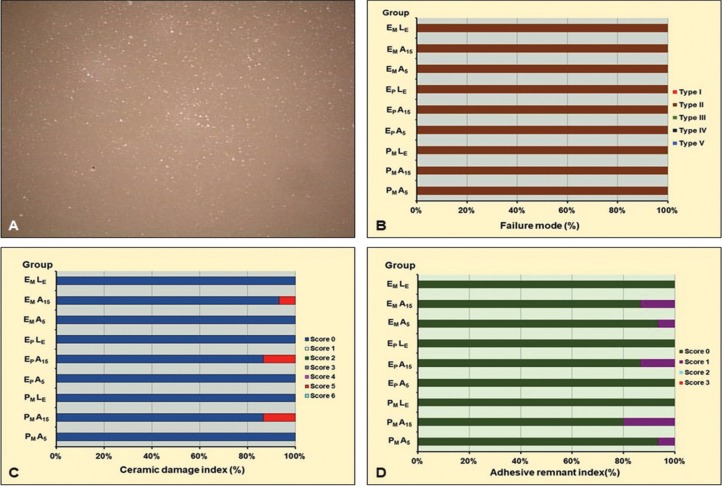
(A) Stereo-micrograph of debond surface of ceramics at 10X magnification, (B) indicated adhesive mode of failure (FM) at adhesive resin-ceramic interface, with (C) difference in pattern of ceramic damage index (CDI), and adhesive resin remnant index (ARI).

The stereo-micrograph of de-bond ceramic surface exhibited a similar pattern of adhesive bond failure, as presented in Figure 2 (A). The ceramic surface was observed to be predominately exposed. Slight remnants of resin adhesive were found on the ceramic surface of groups treated with HF, among which, the groups of A15 tended to demonstrate a slightly higher amount of resin adhesive remnants as compared to A5 groups. The frequency distribution of FM was determined in percentage for each group, as indicated in Figure 2 (B). The patterns of FM, either laser treated or HF etched for 5 or 15 seconds, were mainly demonstrated in Type II, which indicated failure at adhesive resin–ceramic interface. Only ≤ 10% of the bracket base was exposed and ≥ 90% of the de-bonded ceramic was free of adhesive resin. The Chi-square statistics indicated no statistically significant influence on the mode of failure due to the different types of ceramic and methods of ceramic surface treatment (*p* > 0.05). The amount of ceramic damage (%) based on CDI have been indicated in Figure 2 (C). No detectable ceramic surface damage was observed in all groups, except for the groups PFA15, EPA15, and EMA15 that exhibited localized minimal ceramic surface damage. The Chi-square statistics indicated a statistically significant influence on CDI of the method of ceramic surface treatment (*p* < 0.05) but not of the type of ceramic used (*p* > 0.05). The amount of adhesive remnant based on ARI were indicated in Figure 2 (D). The patterns of ARI were quite similar for most groups except for the HF groups, in which, A15 exhibited slightly higher amounts of adhesive remnants on the ceramic surface as compared to the A5 groups. The Chi-square statistics demonstrated a significant difference in ARI among groups (*P* < 0.05). Significant influence on ARI due to ceramic surface treatment (*p* < 0.05) but not for the type of ceramics (*p*>0.05) was suggested.

**Figure 3:**
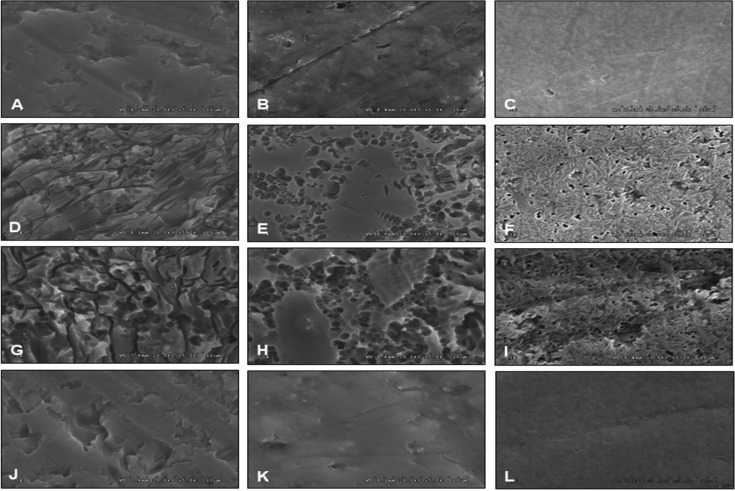
SEM photomicrographs indicated untreated surface (A, B, C), HF-etched surface for 5 seconds (D, E, F), HF-etched surface for 15 seconds (G, H, I), and Er-YAG lased surface (J, K, L) of ceramic veneering metal (A, D, G, I), Empress CAD (B, E, H, K), and e.Max CAD (C, F, I, L) respectively.

The SEM photomicrographs of ceramic surfaces treated with different techniques in comparison to untreated surface at × 5,000 magnifications have been shown in Figure 3. It clearly demonstrates the difference in surface architectures among the groups. The non-treated surface revealed a general smooth surface architecture of the glass, a result of glazing, as depicted in Figure 3 (A–C). The SEM photomicrograph of the HF-etched surface of ceramic revealed generalized irregular surface architectures, as presented in Figure 3 (D–I). Significant higher irregularities of surface were exhibited on A15, as shown in Figure 1 (G-I), as compared to A5, as depicted in Figure 1 (D–F). The crystal structures were exposed on the surface since the glass phase was removed through the etching procedure. The morphology of the laser-treated ceramic specimen exhibited a scaly appearance, as shown in Figure 3 (J–L). The HF treated ceramic surface exhibited higher surface irregularities in comparison to the laser treated surface.

## Discussion

The retention of ceramic brackets on ceramic surface is crucial. In this study, the shear bond strength between ceramic brackets and ceramic material was evaluated in relation to the effect of different methods of ceramic surface treatments and types of ceramics. The results clearly demonstrated that the bond strength of ceramic bracket to ceramic material was significantly affected by the method of treatment on ceramics surface (*p* < 0.05) but not significantly affected by the type of ceramic (*p* > 0.05). Therefore, the null hypothesis was rejected for the method of ceramic surface treatment but accepted for the type of ceramic used. The surface treatment by LE was found to be capable of offering enhanced bond strength than A5 but slightly lesser strength as compared to A15. HF treated ceramic surface form an effective method but may pose the risk of inducing ceramic damage upon de-bonding, a possibility supported by other studies that has made dentists hesitant in using the method (9,17) Er-YAG laser was found to be more appropriate for surface treatment for retaining the ceramic bracket. The laser-treated ceramic surfaces introduced a value of bond strength higher than that offered by the HF-A5 and comparable to that given by HF-A15. This result was consistent with other studies that indicated Er-YAG lasers’ capability in offering suitable bond strength (24,25,27).

However, other factors are influenced with the bond strength of bracket to ceramic, such as architecture and composition of bracket base, surface topography and composition of ceramic material, composition of the resin adhesive, and methods for ceramic surface treatment. In this study, the ceramic bracket base was flat, ensuring the optimal adaptation to the surface of the ceramic specimen without any conditioning upon failure mode. Extremely high bond strength tends to exhibit cohesive failure in ceramic material, which causes ceramic fracture, and sometimes, it exhibits cohesive failure in ceramic bracket, which leaves a significant amount of adhesive resin on the ceramic surface that requires to be cleaned and mostly causes ceramic surface destruction by the finishing bur (5). In this study, all groups exhibited mainly adhesive failures, except the group A15 that occasionally exhibited ceramic ditching upon de-bonding, a result that coincided with earlier studies (5,15). A bond strength higher than 13 MPa tends to cause cohesive failure in ceramic (15). Extremely high bond strength is not usually required for orthodontic treatment. The optimal bond strength that provides a durable bond that can withstand orthodontic and masticatory force during the period of orthodontic treatment requires to be achieved; yet, it should be susceptible to bracket removal by ceramic restoration without damaging to ceramic surface. Suggestions based on scientific investigations for optimal bond strength of orthodontic bracket did not exist in the literature. However, it was suggested that clinically, the bond strength of 6–8 MPa for a metal bracket bonded to enamel is adequately needed (8). However, in order to clarify the benefits of different surface treatments, it is necessary to consider ARI and CDI along with bond strength to establish a suitable bonding regime for bracket bonding, as presented in this study. This investigation revealed that all tested groups exhibited shear bond strengths exceeding clinically acceptable limits. The average shear bonds strength achieved in this study was considerably higher than the clinically required value. However, the de-bonded ceramic surface did not exhibit any ceramic surface damage upon de-bonding, except the groups A15 that suffered some likelihood of ceramic damage during the bracket de-bonding process. The bond strength of bracket to ceramic surface conditioning by A15 was found to be higher than that of LE, and A5 at 95% level of confidence. The groups of A15 were predominately de-bonded in adhesive failure mode and exhibited more adhesive remnants on the ceramic surface compared to others. The resin remnant needs to be eliminated by finishing bur, and in all cases, accidentally destroyed the surface of the ceramic during the finishing process. Thus, the LE and A5 may be better than A15 for cleaning the ceramic surface after the de-bonding process.

Among several methods of ceramic surface treatment, HF-etching method is generally utilized. The glass phase is particularly eliminated, leaving crystal particles exposed, creating a generalized micro-porosity on the surface of the ceramic to facilitate micro-mechanical retention of resin cement (6,18,29). However, the significant amount and extensive micro-porosities created through HF presented on the ceramic surface even after de-bonding, leaving these defects permanently and became the original source of crack initiation in ceramic restoration as well as the source of bacterial deposition. These surface pits still remained on the ceramic surface restoration and needed to be surface finished by polishing with a diamond polishing paste (6). In general, etching ceramic surface prior to bracket bonding was suggested to be performed for 60 seconds or longer. However, previous studies have shown negative effects of etching for 60 seconds or longer, as these created deep and thin irregularities on the ceramic surface, resulting in difficulty for the bonding agent to diffuse into the irregular portion; these can also possibly induce cohesive fracture of ceramic material (4,13,16). The rough surface created by long etching time is also a disadvantage in terms of the difficulty entailed by the need to revitalize the ceramic surface after de-bonding as the surface was extremely rough. In addition, there were reports on high prevalence of fracture of ceramic surface that were deglazed or roughened associated with the de-bonding process (7). Furthermore, the long etching time, the higher ceramic material loss from the surface and higher roughness than desired was exhibited. HF-A15 was selected from this study in order to maintain the integrity of ceramic surface to minimize the surface alteration that can be hardly detected by the naked eye and minimize the polishing process, which can easily recreate the vitalization of the normal ceramic surface after de-bonding. The study indicated that there were significant extensive micro-porosities on the surface treated with A15 as compared to that observed in other methods. Moreover, HF-etched technique is harmful and irritates soft tissue, thus the intra-orally etching process needs to be carefully executed, with an extremely short duration to prevent causing accidental irritation to oral tissue (6). The use of Er:YAG laser for lasing ceramic surface is intended to modify the ceramic surface architecture to facilitate the bonding of the bracket (25,26). The capability of ceramic in absorbing energy from the Er-YAG to produce a scaly appearance on the ceramic surface, providing retention for adhesive resin to retain ceramic bracket (25). In this study, the laser process actually produced a higher bond strength than A5, but the value was comparable with A15. However, no crazing effect was generated on the ceramic after both laser lasing and de-bonding. This contrasts with other studies that indicated laser treated ceramic did not enhance adhesion with resin cement (26). This is probably associated with the differences in ceramic materials and the method of laser-treating procedure. In this study, the laser beam energy, produced from the pulse energy of 200 mJ, pulse rate 20 Hz, power 10 W, in MSP mode at pulse width 100 μs for 20 seconds, was found to be capable of promoting suitable bracket bonding, as supported by others studies (6,8,10).

There are some concerns regarding the effect of lasers on local temperature change that can cause internal damage to tooth structures. However, a recent study indicated no evidence of pulpal injury unless the temperature was raised beyond the physiologic endurance limits of pulp (30). Raising the temperature by only 0.2 °C was evidenced, a value lower than the threshold for causing pulpal injury. The power adjustment and lasing method involving continuous spraying with water can help regulate the temperature effect. In addition, the laser was used on the ceramic materials that possess low thermal conductivity, acting as an insulator to prevent any thermal effect on pulpal involvement. Ultimately, the ceramic surface’s treatment with Er-YAG for bracket bonding was found to be efficient and time saving. Thus, this study suggests using Er-YAG laser for ceramic surface preparation for bonding ceramic bracket in clinical practice.

Conclusions

The optimal bond strength required for ceramic bracket bonding to ceramic restoration should be adequately strong to withstand force from orthodontic treatment, and the bracket should be easily removable without causing damage to ceramic restoration. The Er-YAG laser irradiation provided sufficient bond strength between ceramic brackets and ceramic surfaces and is beneficial for allowing de-bonding process without damaging the ceramic surface, as evidenced by this study. The Er:YAG laser lased ceramic surface forms an appropriate technique and is recommended as an alternative method for ceramic surface treatment for bonding ceramic bracket.

Clinical significance

Optimal bond strength between the bracket and ceramic material is necessary and dependent on the appropriated ceramic surface treatment. Using Er-YAG laser on a pre-conditioning ceramic surface prior to bonding ceramic bracket with adhesive resin presents a feasible procedure and is recommended for clinical practice.
